# Estimation of the Toxicity of Different Substituted Aromatic Compounds to the Aquatic Ciliate *Tetrahymena pyriformis* by QSAR Approach

**DOI:** 10.3390/molecules23051002

**Published:** 2018-04-24

**Authors:** Feng Luan, Ting Wang, Lili Tang, Shuang Zhang, M. Natália Dias Soeiro Cordeiro

**Affiliations:** 1 College of Chemistry and Chemical Engineering, Yantai University, Yantai 264005, China; 18865557652@163.com (T.W.); tangtang20160216@sina.com (L.T.); zs20160903@163.com (S.Z.); 2 LAQV/REQUIMTE, Department of Chemistry and Biochemistry, Faculty of Sciences, University of Porto, 4169-007 Porto, Portugal; ncordeir@fc.up.pt

**Keywords:** substituted aromatic compounds, toxicity, quantitative structure–activity relationship (QSAR), multiple linear regression (MLR), radial basis function neural network (RBFNN)

## Abstract

Nowadays, quantitative structure–activity relationship (QSAR) methods have been widely performed to predict the toxicity of compounds to organisms due to their simplicity, ease of implementation, and low hazards. In this study, to estimate the toxicities of substituted aromatic compounds to *Tetrahymena pyriformis*, the QSAR models were established by the multiple linear regression (MLR) and radial basis function neural network (RBFNN). Unlike other QSAR studies, according to the difference of functional groups (−NO_2_, −X), the whole dataset was divided into three groups and further modeled separately. The statistical characteristics for the models are obtained as the following: MLR: *n* = 36, R^2^ = 0.829, RMS (root mean square) = 0.192, RBFNN: *n* = 36, R^2^ = 0.843, RMS = 0.167 for Group 1; MLR: *n* = 60, R^2^ = 0.803, RMS = 0.222, RBFNN: *n* = 60, R^2^ = 0.821, RMS = 0.193 for Group 2; MLR: *n* = 31 R^2^ = 0.852, RMS = 0.192; RBFNN: *n* = 31, R^2^ = 0.885, RMS = 0.163 for Group 3, respectively. The results were within the acceptable range, and the models were found to be statistically robust with high external predictivity. Moreover, the models also gave some insight on those characteristics of the structures that most affect the toxicity.

## 1. Introduction

With the rapid development of science and technology, tens of thousands of new chemicals are synthesized and widely used in all walks of life every day. However, as we all know, if chemicals are used or handled incorrectly, they may enter the aquatic environment or bio-accumulate in the food chain, where they may adversely impact the people, ultimately. One of the current interests in medicinal chemistry, environmental sciences, and especially for toxicology, is to rank and establish the chemical substances with respect to their potential hazardous effects on humans, wildlife, and aquatic flora and fauna [1]. Among the vast organic matter, it is noteworthy that the substituted aromatic compounds [2,3,4,5,6,7,8] occupy important positions, since they are produced in large quantities and released into the environment as a result of their wide use in agriculture and industry, and are widely distributed in air, natural water, waste water, soil, sediment, and living organics [9,10]. In addition, recent studies have proved that the substituted aromatic compounds are also a kind of biotoxic environmental pollutant, and even have the effects of carcinogenesis and gene mutation on organisms [10,11]. Therefore, studies on the properties of substituted aromatics have important significance.

Up to now, both experimental [12,13,14,15] and theoretical methods [16,17] have been used to evaluate kinds of substituted aromatic compounds for their different toxicities. Also, it is well known that the theoretical predictions of properties or activities by quantitative structure–activity relationship (QSAR) studies have been widely adopted and applied since the 90s, because of their advantages, such as rapidness, easiness, sensitiveness, and cheapness [11]. The QSAR method has been widely applied in different fields, including physical chemistry, pharmaceutical chemistry, environmental chemistry, toxicology, and other research fields [18]. It has been proven that the use of QSAR modeling for toxicological predictions would help to determine the potential adverse effects of chemical entities in risk assessment.

For a long time, a lot of meaningful research focusing on the toxicity of substituted aromatic compounds by QSAR approach have been carried out. In 1982, Schultz et al. tried to perform the QSAR study between the cellular response to *Tetrahymena pyriformis* and molecular connectivity indexes for a series of 24 mono- and dinitrogen heterocyclic compounds. In this study, the authors established a better model than before, and pointed out that toxicity increases with an increase in the number of atoms and degree of methylation per compound, and that toxicity decreases with an increase in nitrogen substitution [1]. In 1998, Cronin et al. established several QSAR models focusing on a dataset of 42 alkyl- and halogen-substituted nitro- and dinitrobenzenes to *Tetrahymena pyriformis* [19]. They found that the nitrobenzenes were thought to elicit their toxic response through multiple (and mixed) mechanisms by one or two molecule descriptor models. In 2001, in order to compare the differences among kinds of QSAR model-building methods, Cronin and Schultz developed QSAR studies for the toxicity of 268 aromatic compounds in the *Tetrahymena pyriformis* growth inhibition assay [16]. In their study, they not only compared the influence of different descriptors on the models, but also the Bayesian regularized neural network (BRANN) and partial least-squares (PLS) analysis to build the models. In the following year, the same authors performed also the same study on a dataset of phenolic toxicity data to *Tetrahymena pyriformis* [17]. The above works gave us some guidelines or directions on how to build better models on the toxicities to this group of compounds. Netzeva et al. developed relative simple QSARs models (one or two descriptors) for the acute toxicity of a dataset of 77 aromatic aldehydes to the ciliate *Tetrahymena pyriformis* using mechanistically interpretable descriptors [20]. They revealed that the octanol/water partition coefficient (log K_OW_) is the most important descriptor, and the models would be improved by using another electronic descriptor. Roy et al. performed a QSAR studies on the toxic potency to *Tetrahymena pyriformis* of a dataset of 174 aromatic compounds (phenols, nitrobenzenes, and benzonitriles) using electrophilicity index [21]. In this study, the compounds in the dataset were divided into the electron donor and acceptor group, and they stated that electrophilicity indices, along with the total Hartree–Fock energy, can be used to build the model perfectively. Later, the performances of the linear and nonlinear models were estimated by Devillers et al. using a structurally heterogeneous set of 200 phenol derivatives on *Tetrahymena pyriformis*. In this study, the authors pointed out the superiority of the nonlinear methods over the linear ones to find complex structure–toxicity relationships among large sets of structurally diverse chemicals [22]. Tetko et al. gave studies on the applicability domain and the influence of the overfitting in the QSAR model building process by the toxicity dataset against *Tetrahymena pyriformis* [23]. The hierarchical technology for QSAR was performed using 95 diverse nitroaromatic compounds against the ciliate *Tetrahymena pyriformis* [24]. Zarei et al. developed a model for the prediction of the toxicity of 268 substituted benzene compounds including phenols, monosubstituted nitrobenzenes, multiply substituted nitrobenzenes and benzonitriles to *T. pyriformis* using bee algorithm (BA) for selecting descriptors and adaptive neuro-fuzzy inference system (ANFIS) for building model [25]. A molecular structural characterization (MSC) method named molecular vertexes correlative index (MVCI) was successfully used to describe the structures of 30 substituted aromatic compounds, and the results suggested good stability and predictability of the QSAR models [26]. Comparative molecular field (CoMFA), molecular similarity index analysis (CoMSIA), and density functional theory (DFT) methods were used to establish QSAR models for analyzing and predicting the toxicities of 31 substituted thiophenols [27]. And later, Salahinejad et al. also used the CoMFA, CoMSIA, and VolSurf techniques to develop valid and predictive models able to estimate the toxicity of substituted benzenes toward *T. pyriformis*. In the paper, they confirmed that in addition to hydrophobic effects, electrostatic and H-bonding interactions also play important roles in the toxicity of substituted benzenes, as well as that the information obtained from CoMFA and CoMSIA 3-D contour maps could be useful to explain the toxicity mechanism of substituted benzenes [28]. The linear (MLR) and nonlinear statistical (RBFNN) methods were used by us to build a reliable, credible, and fast QSAR model for the prediction of mixture toxicity of non-polar narcotic chemicals, including 9 PFCAs, 12 alcohols, and 8 chlorobenzenes and bromobenzenes. The predictive values are in good agreement with the experimental ones [18]. In the same way, recursive neural networks (RNN) and multiple linear regression (MLR) methods were also employed to build models for prediction of the toxicity values of 69 benzene derivatives, both methods provided good results as compared to other studies available in the literature [29]. To build a reliable and predictive QSAR model, a genetic algorithm along with partial least square (GA–PLS) was employed to select the optimal subset of descriptors that significantly contribute to the toxicity of 45 nitrobenzene derivatives to *Tetrahymena pyriformis* [30].

The goal of present study was to develop reliable and predictive QSAR models using both MLR and RBFNN methods to identify and predict the acute toxicity (the 50% growth inhibitory concentration IGC_50_) of substituted aromatic compounds to the aquatic ciliate *Tetrahymena pyriformis*. For this purpose, the whole dataset was divided into three groups with respect to the important function group of the substituted aromatic compounds such as −NO_2_, −X etc. They were Group 1: Compounds with NO_2_ group, etc. (46 compounds); Group 2: Compounds with –X, etc. (75 compounds); Group 3: Compounds with both −NO_2_ and −X, etc. (39 compounds). In so doing, different accurate models were built to evaluate the toxicities of these aromatic compounds.

## 2. Materials and Methods

### 2.1. Datasets

For the aromatic compounds, Wei et al. have mentioned that the order of the contribution of the special substituents to the toxicity of the aromatic compound is: −NO_2_ > −Cl > −CH_3_ > −NH_2_ > −OH [31]. Based on the dataset given by Schultz et al. [32], we selected the typical compounds containing the most influential functional groups (−NO_2_ and –X), and divided them into three subgroups. Group 1 includes 46 compounds whose chemical structures have the functional group −NO_2_ without −X. Among them, 36 compounds were substituted by a −NO_2_ and 10 compounds were substituted by two −NO_2_. Group 2 contains 75 compounds which have functional groups –X without −NO_2_. Among them, the 54, 16, and 5 compounds were replaced by one, two, or three functional groups −X, respectively. Group 3 contains 39 compounds, in which both the −NO_2_ and −X functional groups are included, and the total number of substituents for −NO_2_ and −X is not more than 3.

In this study, compounds in each group were randomly divided into two subsets. One called training set was used to build a model, and there were 36, 60, 31 compounds in the training set for Group 1, 2, 3, respectively. The remaining compounds were used to verify the robustness and feasibility of the model as a test set which includes 10, 15, and 8 for the corresponding groups, respectively. The CAS number, name, and toxicity (−log IGC_50_) of the above compounds are all listed in Table 1.

### 2.2. Molecular Descriptors’ Generation and Selection

To calculate the molecular descriptors of each compound, their structures were drawn using ISIS Draw 2.3 (MDL Information Systems, Inc., San Ramon, CA, USA) [33]. The MM^+^ molecular mechanics forcefield in the HyperChem 6.0 program (Hypercube, Inc.: Waterloo, ON, Canada) was then used to carry out the preliminary molecular geometry optimization [34]. The further optimization of the compound structure was done by semi-empirical PM_3_ method utilizing the Polak–Ribiere algorithm until the root mean square gradient was 0.01 kcal/mol [35]. Finally, a more precise optimization was achieved by MOPAC 6.0 software package (Indiana University: Bloomington, IN, USA) [36]. Afterwards, the final optimized structures were converted to the CODESSA 2.63 program (University of Florida, Gainesville, FL, USA) for calculating the five classes of descriptors, namely constitutional, topological, geometrical, electrostatic, and quantum-chemical descriptors [37]. It was necessary to explain that the logP descriptor, which cannot be calculated by the CODESSA 2.63, but can be obtained by Hyperchem, was then added to the descriptors pool [34]. Through doing these, 494, 597, and 611 descriptors were gained for each of the studied compounds in Group 1, 2, and 3, respectively.

Before establishing the QSAR models, it is necessary to remove the insignificant descriptors, and the constant and highly intercorrelated descriptors (the intercorrelation of the descriptors should be lower than 0.8). In this paper, the heuristic method (HM) was used to achieve a thorough search for the best multilinear correlations with the computed descriptors in the framework of the program CODESSA 2.63 [37].

### 2.3. Multiple Linear Regressions (MLR)

Multiple linear regressions (MLR) are often accepted as a classical method for solving linear problems when there are two or more than two independent variables in QSAR modeling. The purpose of MLR is to find a mathematical function which best depicts the desired activity Y (here, −log IGC_50_ values) as a linear combination of the X-variables (the molecular descriptors), with the regression coefficients *b_n_*. The equation is as follows:

*Y* = *b*_0_ + *b*_1_*x*_1_ +*b*_2_*x*_2_ + … +*b_n_x_n_*.

Usually, the good fit alone does not guarantee that the model is useful for prediction purposes by the *R*^2^ (coefficient of determination), *LOOq*^2^ (leave-one-out correlation coefficient), *RMS* (root mean square error), *F* (Fisher’s statistics), etc. [38]. Some statistical characteristics of the test set are also needed to be considered: *R*^2^ (coefficient of determination), $R_{0}^{2}$ (the coefficients of determination, predicted vs observed activities, when the Y-intercept *b*_0_ is set to zero), as well as by their corresponding slopes *k* and *k*′. The following conditions need to be fulfilled to adequately estimate the predictive ability of a model [39]:

$$q^{2}>0.5$$

$$R^{2}>0.6$$

$$\frac{\left(R^{2}-R_{0}^{2}\right)}{R^{2}}<0.1\;or\;\frac{\left(R^{2}-R_{0}^{\prime 2}\right)}{R^{2}}<0.1$$

$$0.85\leq k\leq 1.15\;\mathrm{or}\;0.85\leq k^{\prime }\leq 1.15$$

### 2.4. Radial Basis Function Neural Networks (RBFNN)

In general, RBFNN may have a better result than MLR, because it can take into account some nonlinear behavior between the molecular descriptors and the desired activities values (−log IGC_50_). The detailed introduction of RBFNN has been stated in previous studies [40,41], so we only make a simple statement of the key parts here.

The RBFNN is a typical feed forward neural network which is composed of three layers, which are the input layer, the hidden layer, and the output layer. The first layer is linear, and distributes the input values, while the next layer is nonlinear, and uses radial basis function. The third layer linearly combines the outputs. Each neuron in each layer is adequately linked to the next layer. However, there is no connection between neurons in a given layer. Each hidden layer unit stands for a single radial basis function, which is characterized by a center and a width. In this layer, each neuron uses a radial basis function as nonlinear transfer function to handle the input information from the previous layer. The most common use of RBF is the Gauss function, characterized by the center (*c_j_*) and width (*r_j_*) [42]. It is used to measure the Euclidean distance between the input vector (*x*) and the radial basis function center (*c_j_*), and gain the nonlinear transformation within the hidden layer, defined as

$$h_{j}=exp\left(-{\left\|x-c_{j}\right\|}^{2}/r_{j}^{2}\right),$$

where *h_j_* is the output of the *j*th RBF unit, while *c_j_* and *r_j_* are the center and width of such a unit, respectively. And the operation of the output layer is linear and is given by

$$y_{k}\left(x\right)=\overset{n_{h}}{\underset{j=1}{\sum }}w_{kj}h_{j}\left(x\right)+b_{k}$$

where *y_k_* is the *k*th output unit for the input vector *X*, *w_kj_* is the weight connection between the *k*th output unit and the *j*th hidden layer unit, and *b_k_* is the respective bias.

In the present study, we used the MATLAB package (MathWorks, Natick, MA, USA) (www.mathworks.com/products/matlab/) to accomplish all the RBFNN calculations. The total functions of the RBFNN model can be evaluated by the same statistical parameters as the MLR method together with its reliability and robustness.

### 2.5. Applicability Domain (AD) of the Model

It is necessary to give the application domain (AD) of the model. The applicability domain (AD) of a QSAR model refers to a theoretical region in the space defined by the compounds in the training set. It demonstrates the nature of the compound molecules that can be utilized in the built model. That is to say, AD restricts a theoretical region, also for unknown chemicals without experimental data, with the lowest number of bad predictions (Y-outliers) and chemicals far from the training structural domain [43]. In this study, a William’s plot, i.e., a plot of standardized residuals (R) vs leverages was used [44]. Here, a simple measure of a chemical being too far from the applicability domain of the model is its leverage, *h_i_* [43], as follows:

$$h_{i}=x_{i}^{T}{\left(x^{T}x\right)}^{-1}x_{i}\left(i=1,2,\cdots ,n\right).$$

In the above equation, *x_i_* represents the descriptor row vector of the studied compound, while *x* represents the *n* × *k* − 1 matrix of *k* model descriptor values for the *n* training set compounds. The superscript “*T*” refers to the transpose of the matrix/vector. *h_i_* characterizes the leverage of a compound, and is one of the coordinates of the William’s plot (standardized residuals versus leverage).

## 3. Results and Discussion

### 3.1. MLR Results

As mentioned above, based on the structural differences among the molecules which are caused by the influential functional groups (−NO_2_ and −X), Group 1, 2, and 3 have 46, 75, 39 compounds, respectively. The models of each group were established by the training sets. Before doing this, the heuristic method (HM) was used to conduct the descriptor selection. After the preselection of the descriptors, 178, 203, and 160 descriptors were left for each group by removing of the descriptors not obeyed the thumb rules [45].

Multilinear regression models were then developed in a stepwise procedure, that is, the descriptors and correlations were sorted by the values of the *F*-test and the correlation coefficients. Beginning with the top descriptor from the list, two-parameter correlations were calculated. Later, the descriptors were added one by one, until the preselected number of descriptors in the model is fulfilled. Finally, three descriptors were used to describe the relationship between molecule structure and toxicity for each group of compounds. The selected descriptors and their chemical meaning, along with the statistical parameters, are listed in Table 2, Table 3 and Table 4.

**Figure 1:**
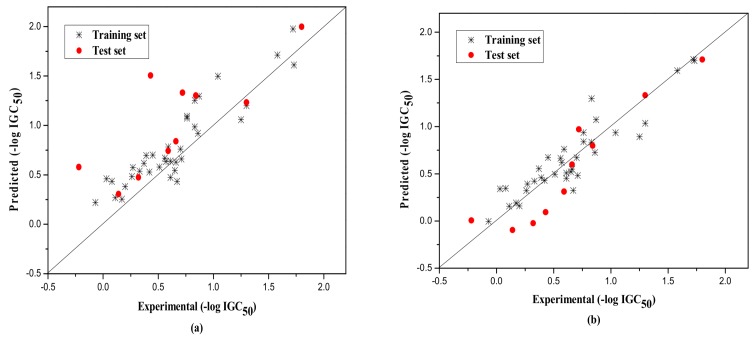
Plot of the predicted versus experimental −log IGC_50_ including the training and the test set compounds of Group 1 by MLR model (**a**) and by RBFNN model (**b**).

**Figure 2:**
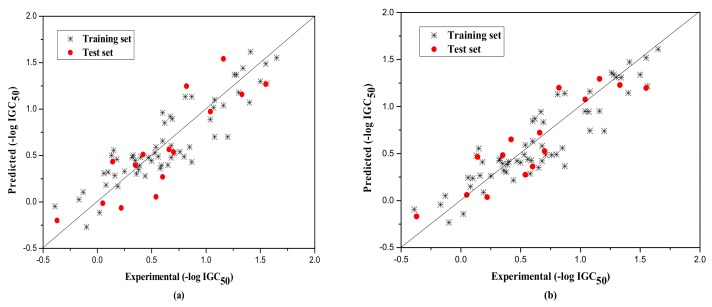
Plot of the predicted versus experimental −log IGC_50_ including the training and the test set compounds of Group 2 by MLR model (**a**) and by RBFNN model (**b**).

**Figure 3:**
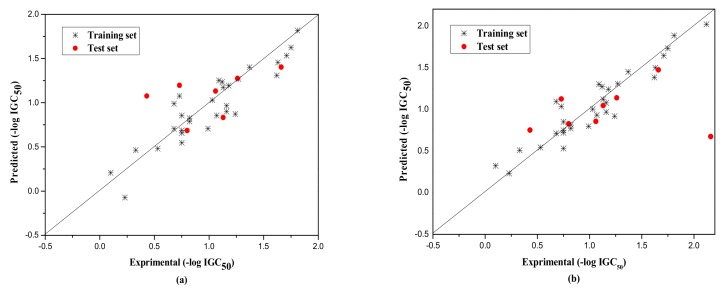
Plot of the predicted versus experimental −log IGC_50_ including the training and the test set compounds of Group 3 by MLR model (**a**) and by RBFNN model (**b**).

The external test set was also used to further evaluate the three models. The statistical parameters obtained are as follows: *N*_ext_ = 10, *R*^2^ = 0.917, $q_{\mathrm{ext}}^{2}$ = 0.851, *F* = 13.820, RMS = 0.222 for group 1; *N*_ext_ = 15, *R*^2^ = 0.789, $q_{\mathrm{ext}}^{2}$ = 0.732, *F* = 13.720, RMS = 0.266 for group 2; *N*_ext_ = 8, *R*^2^ = 0.733, $q_{\mathrm{ext}}^{2}$ = 0.730, *F* = 260.404, RMS = 0.380 for Group 3. Figure 1, Figure 2 and Figure 3a show the predicted vs observed −log IGC_50_ values for all the training and test set compounds. Thus, it can be seen that the model is reasonable in both statistical significance and predictive ability.

### 3.2. Model Applicability Domain Analysis and Improved MLR Model

**Figure 4:**
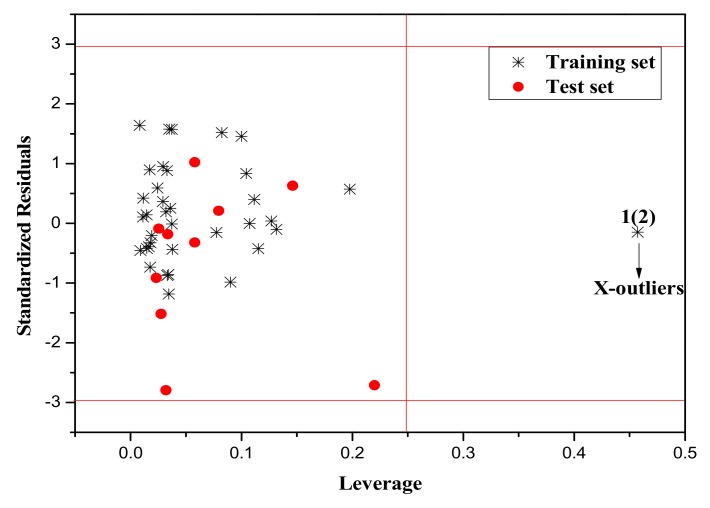
The William’s plot for the training and test set compounds of Group 1 by MLR model.

**Figure 5:**
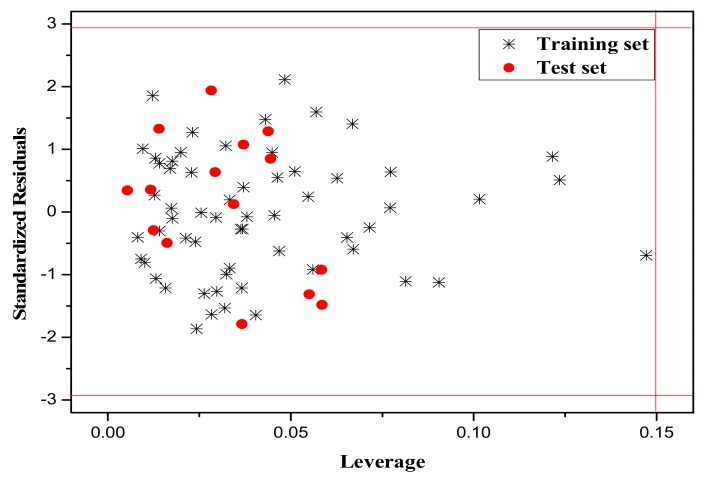
The William’s plot for the training and test set compounds of Group 2 by MLR model.

**Figure 6:**
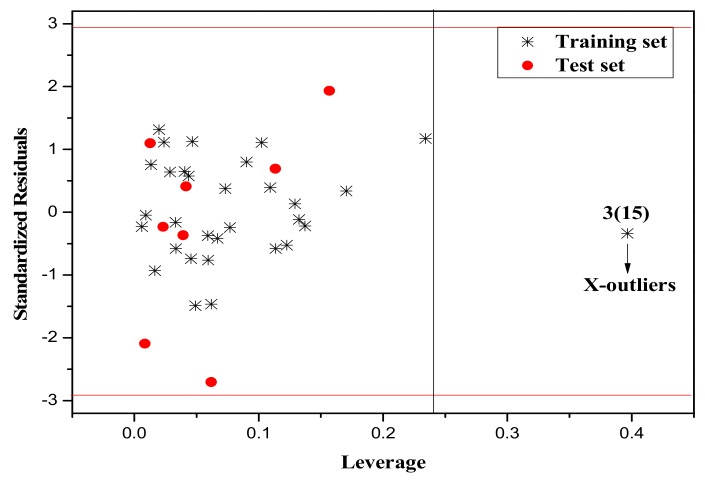
The William’s plot for the training and test set compounds of Group 3 by MLR model.

It is also an important step to consider the possible outliers of the models. In order to visualize the AD, the plot of standardized cross-validated residuals versus leverage (the William’s plot), which can provide an immediate and simple graphical detection, was used to find out the outliers from the models. In this plot, the horizontal and vertical straight lines represent the normal control values of Y-outliers and X-outliers, respectively. The limit of X-coordinate is 3*m*/*n*, where *m* is the number of model parameters, and *n* is the number of samples belonging to the training set. In the present study, the normal control value for Y-outliers (RES) was set as ±3σ. Figure 4, Figure 5 and Figure 6 show the William’s plot based on the MLR models for the whole dataset compounds of group 1, 2, 3, respectively.

As can be judged from Figure 4, in the model for Group 1, there is one X-outlier (for Group 1: compound **2**), which is 2-nitroanisole. In its structure, there are two functional groups, −NO_2_ and methoxy. The former is in all of the compounds belonging to this group as a strong electron-withdrawing group. However, the methoxy group has oxygen lone pair electrons which are a strong electron donor moiety, compared to other ones in the group. Therefore, care should be taken when using the compounds with methoxy, since they can activate the benzene ring and exert an unusual influence on the toxicity. And from Figure 6, it can also be seen that there is a X-outlier (for group 3: compound **15**), that is, 2-chloromethyl-4-nitrophenol. This compound has three electron-withdrawing moieties, including—Cl, −OH, and −NO_2_, which has almost the strongest induction effect of the compounds in this group. Also, there seem to be another outlier (for Group 3: compound **36**), which belongs to the test set. This may be due to variability in the measurement, or it may indicate experimental error.

If the handling of the outliers is unreasonable, the accuracy of the model will be affected. Thus, the quality and ability of the model prediction will be affected. Therefore, we removed the outliers from Group 1 and Group 3, set up the models anew, and the results were as follows: for the training set of Group 1 (removing compound **2** in Group 1): *N* = 35, *R*^2^ = 0.926, LOOq^2^ = 0.916, *F* = 94.266, RMS = 0.125. For the training set of Group 3 (removing compound **15** in Group 3): *N* = 30, *R*^2^ = 0.852, LOOq^2^ = 0.834, *F* = 49.710, RMS = 0.196. The statistical parameters of the model are better after removing the escape values.

To further assess the predictive powers of the model established by the MLR method, parameters such as $\frac{\left(R^{2}-R_{0}^{2}\right)}{R^{2}},k,k^{\prime }$, etc., were also calculated, and the results were shown in Table 5. From the table, we can see the statistical results were all within the acceptable ranges for the methods of MLR.

### 3.3. Validation Results of the Models

Further, a fivefold cross-validation algorithm was applied for validation of the stability of the three models. The members selected for each group (i.e., groups A, B, C, D, and T) were shown in Table 1. The *R*^2^, *F*, and RMS values for each validation along with their average values were shown in Table 6 for the MLR models. As can be seen, both models are stable, judging from the obtained values for the average training quality and for the average predicting quality.

### 3.4. RBFNN Results

In the field of QSAR research, RBFNN often shows better results than MLR because of its ability to consider some nonlinear relationships between the molecular structure and its activity. In order to confirm this view, RBFNN was utilized to build nonlinear predictive models using the same descriptors selected by the MLR models. The RBFNN can be traced as i-n_k_-1 net to indicate the number of units in the three layers, respectively. Meanwhile, the width (r) of RBF was computed by systemically changing its value in the training step from 0.1 to 4.0 with increments of 0.1. For the three groups of compounds belonging to training sets in this study, the RBFNN models were 3-10-1, 3-9-1, and 3-9-1, along with widths of 0.8, 2.0, and 1.7, respectively.

Their statistical results of the training and the test set are as follows. Group1: for training set, *N* = 36, *R*^2^ = 0.843, LOOq^2^ = 0.838, *F* = 182.306, RMS = 0.167, and for the test set, *N*_ext_ = 10, *R*^2^ = 0.881, $q_{\mathrm{ext}}^{2}$ = 0.867, *F* = 59.483, RMS = 0.210; Group 2: for training set, *N* = 60, *R*^2^ = 0.821, LOOq^2^ = 0.818, *F* = 265.898, RMS = 0.192, and for the test set, *N*_ext_ = 15, *R*^2^ = 0.810, $q_{\mathrm{ext}}^{2}$ = 0.796, *F* = 55.506, RMS = 0.232; Group 3: for training set, *N* = 31, *R*^2^ = 0.885, LOOq^2^ = 0.882, *F* = 224.261, RMS = 0.163, and for the test set, *N*_ext_ = 8, *R*^2^ = 0.632, $q_{\mathrm{ext}}^{2}$ = 0.622, *F* = 63.660, RMS = 0.298. The corresponding predicted endpoint values of each compound in each group were shown in Table 1, and the plot of the predicted and experimental values of both training and test set were displayed in Figure 1b, Figure 2b and Figure 3b. Different from the original literature, [35], we selected and classified the original compounds according to the structural characteristics and further modeled, analyzed, and predicted the corresponding toxicity values. The models, thus established, are also more targeted for the particular compounds, and the statistical results of $\frac{\left(R^{2}-R_{0}^{2}\right)}{R^{2}},k,k^{\prime }$, etc., as shown in Table 5 by RBFNN, also indicated the models to be statistically robust with high external predictivity.

### 3.5. Interpretation of Model Descriptors

In order to deepen the understanding of this study, more detailed explanations of the descriptors selected in each group were performed. For group 1, three descriptors were selected in the QSAR model, namely: *G*^2^, P_AB_, and Enn_(C-H)_. The positive sign of them indicated that the −log IGC_50_ values increased with its increase, and vice versa. *G*^2^ refers to gravitation indexes for all bonded pairs of atoms, and it is defined as $G^{2}=\overset{N_{B}}{\underset{\left(i>j\right)}{\sum }}\frac{m_{i}m_{j}}{r_{ij}^{2}}$ [46], where *m_i_* and *m_j_* are the atomic weights of atoms *i* and *j*, *r_ij_* is the interatomic distance, N_b_ is the number of bonds in the molecule. P_o_ belongs to the valency-related descriptors, which relate to the strength of intermolecular bonding interactions and characterize the stability of the molecules, their conformational flexibility and other valency-related properties [47]. E_nn(C-H)_ is Max n–n repulsion for a C–H bond, calculated as follows: Enn(CH)=ZCZHRCH, where *Z_C_* and *Z_H_* are the nuclear (core) charges of atoms *C* and *H*, respectively, and *R_CH_* is the distance between them. This energy describes the nuclear repulsion driven processes in the molecule, and may be related to the conformational (rotational, inversional) changes or atomic reactivity in the molecule [46].

For Group 2, focusing on the compounds without the functional group −NO_2_, but with −X, three descriptors were chosen. That is, Log P, PNSA-2/TMSA, and P_SIGMA._ PNSA-2/TMSA is FNSA-2 fractional PNSA (PNSA-2/TMSA) [Zefirov’s PC], which contributes to the calculation of atomic partial charges to the total molecular solvent-accessible surface area [46]. P_SIGMA_ represents the maximum bond order for a given pair of atomic species in the molecule, its values for a given pair of atomic species in the molecule with the lower limit P_SIGMA_ (min) > 0.1. LogP stands for the solvational characteristic (hydrophobicity of chemicals) because it is closely related to the change in the Gibbs energy of solvation of a solute between two solvents.

For Group 3, three descriptors were selected to build the model, that is Ic, Enn_(C-C)_, and RPCG. The chemical meaning of them can be seen in Table 4. I_c_ is a geometrical descriptor which relates to the atomic masses, the distance of the atomic nucleus from the main rotational axes, which characterizes the mass distribution in the molecule. $\mathrm{Enn}\left(C-C\right)=Z_{C}Z_{C}/R_{C–C}$, where *Z_C_* and *Z_C_* are the nuclear (core) charges of atoms *C*, and *R_C–C_* is the distance between them. This energy describes the nuclear expulsion driven processes in the molecule, and may be related to the conformational (rotational, inversional) changes or atomic reactivity in the molecule [48]. RPCG, relative positive charge, belongs to electrostatic descriptors. From its coefficient, we can find that the relative positive charge of the molecule is negatively related to the endpoint values (−log IGC_50_).

In summary, we found that the repulsion between the two bonds and the local charge on the surface of the molecule appeared in different models, indicating that these two factors have a greater influence on the structure of the compound and should be relatively valued.

## 4. Conclusions

In the present study, the QSAR models were performed on the study of the acute toxicity of substituted aromatic compounds to the aquatic ciliate *Tetrahymena pyriformis* using the MLR and RBFNN methods, and by dividing the whole dataset into three groups based on the most influential functional group (−NO_2_ and −X). Acceptable statistical results for each model indicated their good stability and good predictability. We can also see from the results of the MLR and RBFNN models that the MLR method can establish reasonable models for evaluating the activity of compounds, and the RBFNN method can provide better statistical parameters. Also, the selected descriptors are effective and feasible for evaluating the toxicity of this group of compounds. Lastly, the results of this study provided useful insights on the characteristics of the structures that most affect the toxicity.

## Figures and Tables

**Table 1:** The CAS number, name, experimental −log IGC_50_ values, predicted −log IGC_50_ values and their corresponding residual for the three groups of compounds.

No.	CAS	Name	Experimental −log IGC_50_	Predicted −log IGC_50_				
				MLR	Residual	RBFNN	Residual	Set
**Group 1. Compounds with the functional group −NO_2_.**								
1 *	619-25-0	3-Nitrobenzyl alcohol	−0.22	0.58	0.80	0.01	0.23	T
2	91-23-6	2-Nitroanisole	−0.07	0.22	0.29	0.00	0.07	A
3	99-09-2	3-Nitroaniline	0.03	0.46	0.43	0.34	0.31	B
4	88-74-4	2-Nitroaniline	0.08	0.43	0.35	0.35	0.27	C
5	99-61-6	3-Nitrobenzaldehyde	0.11	0.27	0.16	0.16	0.05	D
6 *	98-95-3	Nitrobenzene	0.14	0.31	0.17	−0.10	−0.24	T
7	552-89-6	2-Nitrobenzaldehyde	0.17	0.25	0.08	0.19	0.02	A
8	555-16-8	4-Nitrobenzaldehyde	0.2	0.38	0.18	0.16	−0.04	B
9	88-72-2	2-Nitrotoluene	0.26	0.48	0.22	0.32	0.06	C
10	704-13-2	3-Hydroxy-4-nitrobenzaldehyde	0.27	0.57	0.30	0.39	0.12	D
11 *	121-89-1	30-Nitroacetophenone	0.32	0.47	0.15	−0.02	−0.34	T
12	42454-06-8	5-Hydroxy-2-nitrobenzaldehyde	0.33	0.54	0.21	0.42	0.09	A
13	89-62-3	4-Methyl-2-nitroaniline	0.37	0.62	0.25	0.55	0.18	B
14	619-50-1	Methyl-4-nitrobenzoate	0.39	0.70	0.31	0.46	0.07	C
15	99-08-1	3-Nitrotoluene	0.42	0.53	0.11	0.43	0.01	D
16 *	5292-45-5	Dimethyl nitroterephthalate	0.43	1.51	1.08	0.09	−0.34	T
17	619-24-9	3-Nitrobenzonitrile	0.45	0.70	0.25	0.67	0.22	A
18	554-84-7	3-Nitrophenol	0.51	0.58	0.07	0.49	−0.02	B
19	83-41-0	1,2-Dimethyl-3-nitrobenzene	0.56	0.67	0.11	0.66	0.10	C
20	119-33-5	4-Methyl-2-nitrophenol	0.57	0.63	0.06	0.62	0.05	D
21 *	99-51-4	1,2-Dimethyl-4-nitrobenzene	0.59	0.74	0.15	0.31	−0.28	T
22	700-38-9	5-Methyl-2-nitrophenol	0.59	0.78	0.19	0.76	0.17	A
23	4920-77-8	3-Methyl-2-nitrophenol	0.61	0.64	0.03	0.51	−0.10	B
24	3011-34-5	4-Hydroxy-3-nitrobenzaldehyde	0.61	0.47	−0.14	0.45	−0.16	C
25	99-99-0	4-Nitrotoluene	0.65	0.54	−0.11	0.52	−0.13	D
26 *	5428-54-6	2-Methyl-5-nitrophenol	0.66	0.84	0.18	0.60	−0.06	T
27	601-89-8	2-Nitroresorcinol	0.66	0.63	−0.03	0.55	−0.11	A
28	88-75-5	2-Nitrophenol	0.67	0.43	−0.24	0.32	−0.35	B
29	99-77-4	Ethyl-4-nitrobenzoate	0.7	0.76	0.06	0.67	−0.03	C
30	555-03-3	3-Nitroanisole	0.71	0.66	−0.05	0.48	−0.23	D
31 *	97-02-9	2,4-Dinitroaniline	0.72	1.33	0.61	0.97	0.25	T
32	616-86-4	4-Ethoxy-2-nitroaniline	0.76	1.09	0.33	0.84	0.08	A
33	99-65-0	1,3-Dinitrobenzene	0.76	1.07	0.31	0.94	0.18	B
34	100-29-8	4-Nitrophenetole	0.83	0.98	0.15	0.84	0.01	C
35	573-56-8	2,6-Dinitrophenol	0.83	1.25	0.42	1.30	0.47	D
36 *	606-22-4	2,6-Dinitroaniline	0.84	1.30	0.46	0.80	−0.04	T
37	603-71-4	1,3,5-Trimethyl-2-nitrobenzene	0.86	0.92	0.06	0.73	−0.13	A
38	121-14-2	2,4-Dinitrotoluene	0.87	1.30	0.43	1.07	0.20	B
39	329-71-5	2,5-Dinitrophenol	1.04	1.50	0.46	0.94	−0.10	C
40	528-29-0	1,2-Dinitrobenzene	1.25	1.06	−0.19	0.89	−0.36	D
41 *	100-25-4	1,4-Dinitrobenzene	1.3	1.23	−0.07	1.33	0.03	T
42	86-00-0	2-Nitrobiphenyl	1.3	1.20	−0.10	1.03	−0.27	A
43	620-88-2	4-Nitrophenyl phenyl ether	1.58	1.71	0.13	1.59	0.01	B
44	69212-31-3	2-(Benzylthio)-3-nitropyridine	1.72	1.98	0.26	1.71	−0.01	C
45	534-52-1	4,6-Dinitro-2-methylphenol	1.73	1.61	−0.12	1.70	−0.03	D
46 *	4097-49-8	4-(*tert*)-Butyl-2,6-dinitrophenol	1.8	2.00	0.20	1.71	−0.09	T
**Group 2. Compounds with the functional group −X.**								
1 *	348-54-9	2-Fluoroaniline	−0.37	-0.20	0.17	−0.17	0.20	T
2	95-51-2	2-Chloroaniline	−0.17	0.03	0.20	−0.04	0.13	A
3	108-90-7	Chlorobenzene	−0.13	0.11	0.24	0.05	0.18	B
4	372-19-0	3-Fluoroaniline	−0.1	−0.27	−0.17	−0.23	−0.13	C
5	371-41-5	4-Fluorophenol	0.02	−0.12	−0.14	−0.14	−0.16	D
6 *	106-47-8	4-Chloroaniline	0.05	−0.01	−0.06	0.06	0.01	T
7	100-44-7	Benzyl chloride	0.06	0.31	0.25	0.24	0.18	A
8	108-86-1	Bromobenzene	0.08	0.18	0.10	0.15	0.07	B
9	18982-54-2	2-Bromobenzyl alcohol	0.1	0.32	0.22	0.24	0.14	C
10	95-88-5	4-Chlororesorcinol	0.13	0.50	0.37	0.48	0.35	D
11 *	156-41-2	2-(4-Chlorophenyl)-ethylamine	0.14	0.43	0.29	0.46	0.32	T
12	873-63-2	3-Chlorobenzyl alcohol	0.15	0.56	0.41	0.55	0.40	A
13	104-86-9	4-Chlorobenzylamine	0.16	0.28	0.12	0.27	0.11	B
14	615-65-6	2-Chloro-4-methylaniline	0.18	0.46	0.28	0.41	0.23	C
15	367-12-4	2-Fluorophenol	0.19	0.17	−0.02	0.09	−0.10	D
16 *	108-42-9	3-Chloroaniline	0.22	−0.07	−0.29	0.04	−0.18	T
17	873-76-7	4-Chlorobenzyl alcohol	0.25	0.33	0.08	0.26	0.01	A
18	1875-88-3	4-Chlorophenethyl alcohol	0.32	0.48	0.16	0.43	0.11	B
19	95-56-7	2-Bromophenol	0.33	0.50	0.17	0.45	0.12	C
20	95-69-2	4-Chloro-2-methylaniline	0.35	0.45	0.10	0.39	0.04	D
21 *	615-43-0	2-Iodoaniline	0.35	0.40	0.05	0.48	0.13	T
22	591-50-4	Iodobenzene	0.36	0.30	−0.06	0.32	−0.04	A
23	87-60-5	3-Chloro-2-methylaniline	0.38	0.35	−0.03	0.30	−0.08	B
24	95-74-9	3-Chloro-4-methylaniline	0.39	0.39	0.00	0.38	−0.01	C
25	104-88-1	4-Chlorobenzaldehyde	0.4	0.48	0.08	0.41	0.01	D
26 *	103-63-9	(2-Bromoethyl)-benzene	0.42	0.51	0.09	0.65	0.23	T
27	5922-60-1	2-Amino-5-chlorobenzonitrile	0.44	0.28	−0.16	0.22	−0.22	A
28	106-38-7	4-Bromotoluene	0.47	0.48	0.01	0.42	−0.05	B
29	95-79-4	5-Chloro-2-methylaniline	0.5	0.44	−0.06	0.40	−0.10	C
30	95-50-1	1,2-Dichlorobenzene	0.53	0.53	0.00	0.49	−0.04	D
31 *	106-48-9	4-Chlorophenol	0.54	0.06	−0.48	0.27	−0.27	T
32	615-74-7	2-Chloro-5-methylphenol	0.54	0.59	0.05	0.59	0.05	A
33	554-00-7	2,4-Dichloroaniline	0.56	0.49	−0.07	0.44	−0.12	B
34	95-82-9	2,5-Dichloroaniline	0.58	0.36	−0.22	0.29	−0.29	C
35	7120-43-6	5-Chloro-2-hydroxybenzamide	0.59	0.39	−0.20	0.43	−0.16	D
36 *	623-12-1	4-Chloroanisole	0.6	0.27	−0.33	0.36	−0.24	T
37	6627-55-0	2-Bromo-4-methylphenol	0.6	0.96	0.36	0.84	0.24	A
38	16532-79-9	4-Bromophenyl acetonitrile	0.6	0.66	0.06	0.63	0.03	B
39	2973-76-4	5-Bromovanillin	0.62	0.85	0.23	0.87	0.25	C
40	626-01-7	3-Iodoaniline	0.65	0.40	−0.25	0.35	−0.30	D
41 *	140-53-4	4-Chlorobenzyl cyanide	0.66	0.57	−0.09	0.72	0.06	T
42	1585-07-5	1-Bromo-4-ethylbenzene	0.67	0.92	0.25	0.94	0.27	A
43	106-37-6	1,4-Dibromobenzene	0.68	0.61	−0.07	0.58	−0.10	B
44	106-41-2	4-Bromophenol	0.68	0.48	−0.20	0.42	−0.26	C
45	1124-04-5	2-Chloro-4,5-dimethylphenol	0.69	0.90	0.21	0.83	0.14	D
46 *	1570-64-5	4-Chloro-2-methylphenol	0.7	0.54	−0.16	0.52	−0.18	T
47	626-43-7	3,5-Dichloroaniline	0.71	0.52	−0.19	0.49	−0.22	A
48	65262-96-6	3-Chloro-5-methoxyphenol	0.76	0.54	−0.22	0.49	−0.27	B
49	59-50-7	4-Chloro-3-methylphenol	0.8	0.49	−0.31	0.49	−0.31	C
50	2905-69-3	Methyl-2,5-dichlorobenzoate	0.81	1.13	0.32	1.13	0.32	D
51 *	14548-45-9	4-Bromophenyl-3-pyridyl ketone	0.82	1.25	0.43	1.20	0.38	T
52	540-38-5	4-Iodophenol	0.85	0.59	−0.26	0.56	−0.29	A
53	108-43-0	3-Chlorophenol	0.87	0.43	−0.44	0.37	−0.50	B
54	108-70-3	1,3,5-Trichlorobenzene	0.87	1.13	0.26	1.14	0.27	C
55	120-83-2	2,4-Dichlorophenol	1.04	0.89	−0.15	0.95	−0.09	D
56 *	874-42-0	2,4-Dichlorobenzaldehyde	1.04	0.97	−0.07	1.08	0.04	T
57	95-75-0	3,4-Dichlorotoluene	1.07	1.02	−0.05	0.95	−0.12	A
58	120-82-1	1,2,4-Trichlorobenzene	1.08	1.10	0.02	1.16	0.08	B
59	14143-32-9	4-Chloro-3-ethylphenol	1.08	0.70	−0.38	0.74	−0.34	C
60	2374-05-2	4-Bromo-2,6-dimethylphenol	1.16	1.04	−0.12	0.95	−0.21	D
61 *	1689-84-5	3,5-Dibromo-4-hydroxybenzonitrile	1.16	1.54	0.38	1.29	0.13	T
62	88-04-0	4-Chloro-3,5-dimethylphenol	1.2	0.70	−0.50	0.74	−0.46	A
63	90-90-4	4-Bromobenzophenone	1.26	1.37	0.11	1.36	0.10	B
64	7530-27-0	4-Bromo-6-chloro-2-cresol	1.28	1.37	0.09	1.34	0.06	C
65	636-30-6	2,4,5-Trichloroaniline	1.3	1.18	−0.12	1.31	0.01	D
66 *	5798-75-4	Ethyl-4-bromobenzoate	1.33	1.16	−0.17	1.23	−0.10	T
67	13608-87-2	20,30,40-Trichloroacetophenone	1.34	1.44	0.10	1.31	−0.03	A
68	615-58-7	2,4-Dibromophenol	1.4	1.07	−0.33	1.15	−0.25	B
69	88-06-2	2,4,6-Trichlorophenol	1.41	1.62	0.21	1.47	0.06	C
70	134-85-0	4-Chlorobenzophenone	1.5	1.30	−0.20	1.34	−0.16	D
71 *	1016-78-0	3-Chlorobenzophenone	1.55	1.27	−0.28	1.20	−0.35	T
72	90-60-8	3,5-Dichlorosalicylaldehyde	1.55	1.49	−0.06	1.52	−0.03	A
73	591-35-5	3,5-Dichlorophenol	1.56	1.28	−0.28	1.22	−0.34	B
74	90-59-5	3,5-Dibromosalicylaldehyde	1.65	1.55	−0.10	1.61	−0.04	C
75	456-47-3	3-Fluorobenzyl alcohol	−0.39	−0.05	0.34	−0.09	0.30	D
**Group 3. Compounds with both −NO_2_ and −X.**								
1 *	89-59-8	4-Chloro-2-nitrotoluene	0.43	1.08	0.65	0.75	0.32	T
2	585-79-5	1-Bromo-3-nitrobenzene	0.53	0.48	−0.05	0.54	0.01	A
3	7149-70-4	2-Bromo-5-nitrotoluene	0.68	0.99	0.31	1.09	0.41	B
4	100-14-1	4-Nitrobenzyl chloride	0.68	0.70	0.02	0.71	0.03	C
5	610-78-6	4-Chloro-3-nitrophenol	0.73	1.08	0.35	1.03	0.30	D
6 *	7147-89-9	4-Chloro-6-nitro-3-cresol	0.73	1.20	0.47	1.12	0.39	T
7	364-74-9	2,5-Difluoronitrobenzene	0.75	0.66	−0.09	0.85	0.10	A
8	6361-21-3	2-Chloro-5-nitrobenzaldehyde	0.75	0.86	0.11	0.75	0.00	B
9	83-42-1	2-Chloro-6-nitrotoluene	0.75	0.55	−0.20	0.53	−0.22	C
10	88-73-3	1-Chloro-2-nitrobenzene	0.75	0.69	−0.06	0.72	−0.03	D
11 *	121-73-3	1-Chloro-3-nitrobenzene	0.8	0.69	−0.11	0.83	0.03	T
12	87-65-0	2,6-Dichlorophenol	0.82	0.83	0.01	0.81	−0.01	A
13	121-87-9	2-Chloro-4-nitroaniline	0.82	0.79	−0.03	0.77	−0.05	B
14	577-19-5	1-Bromo-2-nitrobenzene	0.99	0.71	−0.28	0.80	−0.19	C
15	2973-19-5	2-Chloromethyl-4-nitrophenol	1.03	1.03	0.00	1.00	−0.03	D
16 *	78056-39-0	4,5-Difluoro-2-nitroaniline	1.06	1.13	0.07	0.86	−0.20	T
17	350-30-1	3-Chloro-4-fluoronitrobenzene	1.07	0.86	−0.21	0.93	−0.14	A
18	42087-80-9	Methyl-4-chloro-2-nitrobenzoate	1.09	1.25	0.16	1.30	0.21	B
19	611-06-3	2,4-Dichloronitrobenzene	1.12	1.23	0.11	1.27	0.15	C
20	51-28-5	2,4-Dinitrophenol	1.13	1.17	0.04	1.12	−0.01	D
21 *	3209-22-1	2,3-Dichloronitrobenzene	1.13	0.83	−0.30	1.04	−0.09	T
22	3819-88-3	1-Fluoro-3-iodo-5-nitrobenzene	1.16	0.90	−0.26	0.97	−0.19	A
23	618-62-2	3,5-Dichloronitrobenzene	1.16	0.96	−0.20	1.08	−0.08	B
24	89-61-2	2.5-Dichloronitrobenzene	1.18	1.19	0.01	1.24	0.06	C
25	99-54-7	3,4-Dichloronitrobenzene	1.24	0.87	−0.37	0.92	−0.32	D
26 *	2683-43-4	2,4-Dichloro-6-nitroaniline	1.26	1.28	0.02	1.14	−0.12	T
27	3460-18-2	2,5-Dibromonitrobenzene	1.27	1.27	0.00	1.31	0.04	A
28	827-23-6	2,4-Dibromo-6-nitroaniline	1.37	1.40	0.03	1.45	0.08	B
29	6641-64-1	4,5-Dichloro-2-nitroaniline	1.62	1.31	−0.31	1.38	−0.24	C
30	609-89-2	2,4-Chloro-6-nitrophenol	1.63	1.46	−0.17	1.50	−0.13	D
31 *	305-85-1	2,6-Iodo-4-nitrophenol	1.66	1.40	−0.26	1.47	−0.19	T
32	3531-19-9	6-Chloro-2,4-dinitroaniline	1.71	1.53	−0.18	1.64	−0.07	A
33	1817-73-8	2-Bromo-4,6-dinitroaniline	1.75	1.63	−0.12	1.73	−0.02	B
34	97-00-7	1-Chloro-2,4-dinitrobenzene	1.81	1.82	0.01	1.88	0.07	C
35	709-49-9	2,4-Dinitro-1-iodobenzene	2.12	1.78	−0.34	2.02	−0.10	D
36 *	70-34-8	2,4-Dinitro-1-fluorobenzene	2.16	1.67	−0.49	0.67	−1.49	T
37	350-46-9	1-Fluoro-4-nitrobenzene	0.1	0.21	0.11	0.32	0.22	A
38	1493-27-2	1-Fluoro-2-nitrobenzene	0.23	-0.07	−0.30	0.23	0.00	B
39	100-00-5	1-Chloro-4-nitrobenzene	0.33	0.46	0.13	0.51	0.18	C

* represents the compound in the test set.

**Table 2:** Descriptors, Coefficients, Standard Error, and *t*-Test Values for the Best MLR Model of Group 1.

	Coefficients	Standard Errors	*t*-Test	Descriptors
**0**	73.123	20.547	3.559	Intercept
**1**	0.002	0.000	10.075	Gravitation index (all bonds) (G^2^)
**2**	−1.016	0.188	−5.394	Max bond order of a O atom (P_o_)
**3**	−1.082	0.510	−3.568	Max n–n repulsion for a C–H bond (Enn(C–H))
*N* = 36, *R*^2^ = 0.829, LOOq^2^ = 0.813, *F* = 51.697, RMS = 0.192				

**Table 3:** Descriptors, Coefficients, Standard Errors, and *t*-Test Values for the Best MLR Model of Group 2.

	Coefficients	Standard Errors	*t*-Test	Descriptors
**0**	−18.030	3.703	−4.869	Intercept
**1**	0.438	0.045	9.808	LogP
**2**	−6.605	0.605	−10.918	FNSA-2 Fractional PNSA (PNSA-2/TMSA) [Zefirov’s PC] (PNSA-2/TMSA)
**3**	17.066	3.794	4.498	Max SIGMA–SIGMA bond order(P_SIGMA_)
*N* = 60, *R*^2^ = 0.803, LOOq^2^ = 0.792, *F* = 76.016, RMS = 0.222				

**Table 4:** Descriptors, Coefficients, Standard Errors, and *t*-Test Values for the Best MLR Model of Group 3.

	Coefficients	Standard Errors	*t*-Test	Descriptors
**0**	−16.640	3.092	−5.382	Intercept
**1**	−2.141	0.322	−6.650	Principal moment of inertia C (Ic)
**2**	0.151	0.024	6.292	Max e–e repulsion for a C–C bond (Enn(C–C))
**3**	−51.290	7.493	−6.845	RPCG Relative positive charge (QMPOS/QTPLUS) [Quantum-Chemical PC] (QMPOS/QTPLUS)
*N* = 31, *R*^2^ = 0.852, LOOq^2^ = 0.835, *F* = 51.678, RMS = 0.193				

**Table 5:** The statistical results of the external test set for the three models of each group.

	Group 1		Group 2		Group 3	
	MLR	RBFNN	MLR	RBFNN	MLR	RBFNN
*R* ^2^	0.83	0.84	0.80	0.82	0.85	0.89
$q_{\mathrm{ext}}^{2}$	0.92	0.88	0.79	0.81	0.73	0.63
$R_{0}^{2}$	0.81	0.84	0.79	0.82	0.84	0.88
$\frac{\left(R^{2}-R_{0}^{2}\right)}{R^{2}}$	0.024	0.00	0.012	0.00	0.012	0.011
*k*	0.88	0.86	0.80	0.83	0.85	0.87
*k*′	1.11	0.97	0.93	0.91	0.93	0.98

**Table 6:** Validation of the MLR models.

Training Set	*R* ^2^	*F*	RMS	Test Set	*R* ^2^	*F*	RMS
Group 1							
A + B + C + D	0.829	51.697	0.192	T	0.917	13.820	0.222
A + B + C + T	0.735	30.457	0.261	D	0.874	48.592	0.153
B + C + D + T	0.742	31.565	0.255	A	0.795	27.199	0.217
A + C + D + T	0.723	28.775	0.259	B	0.889	55.784	0.173
A + B + D + T	0.744	31.912	0.247	C	0.835	35.501	0.217
Average	0.755	34.881	0.243		0.862	36.180	0.196
Group 2							
A + B + C + D	0.803	76.016	0.222	T	0.852	51.678	0.193
A + B + C + T	0.802	75.661	0.225	D	0.748	38.559	0.256
B + C + D + T	0.784	67.805	0.235	A	0.857	78.133	0.193
A + C + D + T	0.786	68.459	0.233	B	0.818	58.416	0.221
A + B + D + T	0.780	66.184	0.235	C	0.831	63.981	0.218
Average	0.791	70.834	0.230		0.821	58.153	0.216
Group 3							
A + B + C + D	0.789	13.720	0.260	T	0.733	260.404	0.380
A + B + C + T	0.760	29.502	0.263	D	0.927	63.850	0.115
B + C + D + T	0.755	27.787	0.254	A	0.899	53.612	0.170
A + C + D + T	0.762	28.869	0.250	B	0.917	66.385	0.159
A + B + D + T	0.754	27.573	0.248	C	0.846	32.957	0.237
Average	0.764	25.490	0.255		0.864	95.442	0.212
